# Safety and efficacy of left-sided three-port laparoscopic gastric cancer surgery: a prospective observational study

**DOI:** 10.3389/fonc.2025.1627001

**Published:** 2025-09-08

**Authors:** Xuan Fang, Ziyang Li, Xiaopeng Gao, Xin Guo, Gang Ji, Yanyang Song, Jiangpeng Wei

**Affiliations:** ^1^ Yuncheng Central Hospital affiliated to Shanxi Medical University, Department of Gastrointestinal Surgery, Yuncheng, Shanxi, China; ^2^ Department of General Surgery, Tianji Hospital, Changzhi, Shanxi, China; ^3^ Department of General Surgery, Heji Hospital, Changzhi, Shanxi, China; ^4^ The First Affiliated Hospital of the Air Force Medical University 986 Hospital, Department of Gastrointestinal Surgery, Xi’an, China; ^5^ The First Affiliated Hospital of the Air Force Medical University, Department of Gastrointestinal Surgery, Xi’an, China

**Keywords:** gastrectomy, laparoscopy, reduced port surgery, stomach neoplasm, survival

## Abstract

**Background:**

While reduced-port laparoscopic gastrectomy(RPLG) has emerged as a minimally invasive alternative, its standardization and long-term efficacy remain underexplored. This study evaluates the comparative outcomes of three-port (TPLDG) versus five-port laparoscopic distal gastrectomy (FPLDG).

**Methods:**

This prospective multicenter study enrolled 355 gastric cancer patients meeting selection criteria. Surgical procedures adhered to D2 lymphadenectomy guidelines, with TPLDG utilizing a left-sided approach without auxiliary ports. Primary endpoints included inflammatory markers, recovery parameters, and 3-year survival outcomes.

**Results:**

The operative outcomes showed comparable results between groups, with similar operative times [140(125,160) *vs.* 135(120,150) minutes, *p*=0.068)] and total lymph node retrieved [(22(19,27) *vs.* 22(18,27) nodes, *p*=0.696)]. Notably, the TPLDG group demonstrated significant recovery advantages, including earlier flatus [(2(2,3) *vs.*3(2,3) days, *p*<0.001)], shorter hospital stays [4(3,5) *vs.* 5.2(4.2,6.3) days, *p*<0.001)], and reduced inflammatory responses as evidenced by lower postoperative CRP [(48.2 ± 21.4) *vs.* (68.5 ± 25.6) mg/L, *p*<0.01)] and IL-6 levels [(82.3 ± 31.2) *vs.* (115.4 ± 38.5)pg/mL, *p*<0.01)]. Importantly, oncological outcomes remained equivalent between groups, with comparable 3-year disease-free survival (85.4% vs 85.8%, *p*=0.85) and overall survival rates (89.4% vs. 89.2%, *p*=0.70), which were consistently maintained across stage-stratified analyses.

**Conclusion:**

TPLDG achieves comparable oncological outcomes to conventional FPLDG while offering significant advantages in postoperative recovery and inflammatory response reduction. The left-sided three-port technique represents a viable standardized approach for RPLG, particularly suited for D2 lymphadenectomy in Asian populations.

## 1 Introduction

Gastric cancer (GC) ranks as the fifth most common malignancy worldwide and the third leading cause of cancer-related deaths (1). Currently, surgical resection remains the cornerstone of treatment, with continuous advancements aimed at enhancing postoperative recovery and oncological outcomes (2). Since its introduction in 1994, laparoscopic gastrectomy has gained widespread acceptance and become a mainstream surgical option for GC (3). In China, several randomized controlled trials have confirmed the safety and efficacy of laparoscopic D2 lymphadenectomy for locally advanced GC, demonstrating its advantages in promoting faster recovery (4, 5). Moreover, studies indicate that laparoscopic gastrectomy offers multiple benefits over open surgery, even in advanced GC following neoadjuvant therapy, with its safety and effectiveness well validated (6).

With the growing emphasis on precision medicine, surgeons are increasingly focused on tailoring surgical strategies to individual patients to achieve optimal outcomes. In recent years, efforts have been made to minimize surgical trauma. Unlike conventional multiport laparoscopic surgery, reduced-port laparoscopic gastrectomy (RPLG)-a less invasive approach with fewer incisions-has emerged (7, 8). Multiple studies suggest that RPLG yields comparable postoperative and oncological outcomes, including 5-year overall survival rates, to traditional multiport laparoscopic gastrectomy, while offering superior cosmetic satisfaction and improved oral intake (9, 10). However, RPLG presents a steeper learning curve, limiting its widespread adoption, and the techniques remain non-standardized. Most current RPLG procedures require additional ports or auxiliary instruments, which may exacerbate the “chopstick effect” and “tailing effect,” prolonging the learning curve and increasing surgical costs (11–13).

Given that most laparoscopic gastrectomies are performed from the left-sided approach and D2 lymphadenectomy remains the standard for advanced GC, our preliminary study validated the feasibility and short-term safety of a three-port laparoscopic gastrectomy with left-sided positioning (14). This approach eliminates the need for extra instruments while enabling both D1+ and D2 lymphadenectomy. To further evaluate its safety and long-term efficacy, we analyzed data from a prospective multicenter observational study, aiming to advance the development of reduced-port laparoscopic gastrectomy.

## 2 Materials and methods

### 2.1 Inclusion criteria

Patients meeting the following criteria were enrolled:

1. Aged 18–75 years; 2. Body mass index (BMI) between 17–24 kg/m²; 3. Preoperative pathological confirmation of primary gastric adenocarcinoma located in the middle or lower third of the stomach; 4. Preoperative assessment indicating a tumor diameter ≤5 cm; 5. No evidence of distant metastasis (e.g., liver, lung) on contrast-enhanced CT; 6. Eastern Cooperative Oncology Group (ECOG) performance status of 0-1; 7. Clinical stage cT1-4aN0-3M0 (AJCC 8th edition TNM staging) with planned R0 resection via totally laparoscopic radical distal gastrectomy; 8. Early GC deemed unsuitable for endoscopic resection upon preoperative evaluation, or patients/families opting against endoscopic treatment.

### 2.2 Exclusion criteria

**Figure 1:**
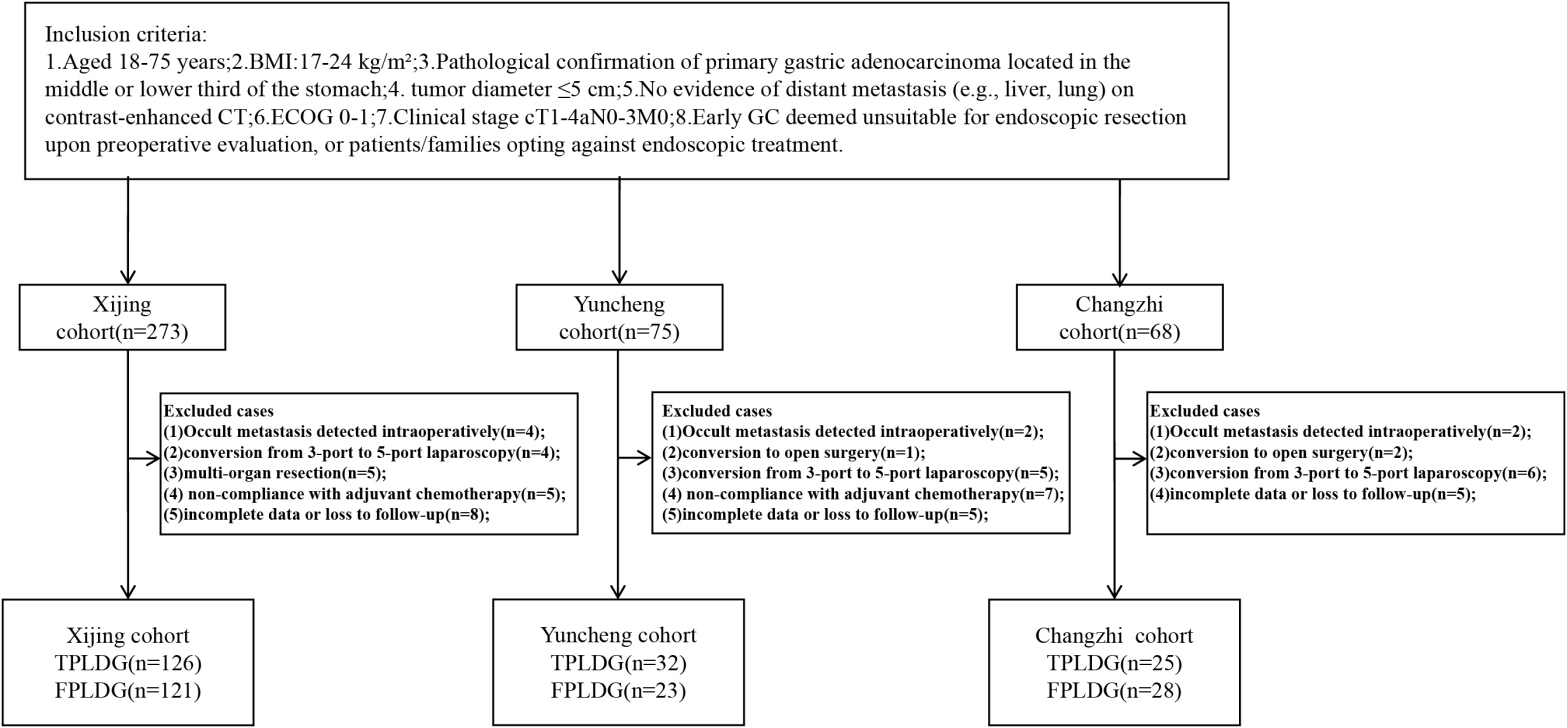
Flow chart of recruiting patients.

Patients were excluded if they met any of the following: 1. Previous upper abdominal surgery (except cholecystectomy) or requirement for multi-organ resection; 2. Conversion to five-port laparoscopic distal gastrectomy (FPLDG) or open surgery during three-port totally laparoscopic distal gastrectomy (TPLDG); 3. Emergency surgery due to complications (bleeding, perforation, obstruction); 4. Non-compliance with postoperative adjuvant chemotherapy; 5. Severe systemic comorbidities contraindicating laparoscopic surgery (additional criteria detailed in the clinical trial registry). This study was approved by the Medical Ethics Committee of the First Affiliated Hospital of Air Force Medical University (Approval No. KY20212225-C-1). All participants provided written informed consent. The trial was registered at www.chictr.org.cn (ChiCTR2200060322). By April 2024, 416 patients were initially enrolled. Exclusions included: 8 cases: occult metastasis detected intraoperatively; 3 cases: conversion to open surgery; 15 cases: conversion from 3-port to 5-port laparoscopy; 5 cases: multi-organ resection; 12 cases: non-compliance with adjuvant chemotherapy; 18 cases: incomplete data or loss to follow-up; Final cohort: 355 patients (see **Figure 1** for recruitment flowchart).

### 2.3 Trocar placement

For patients in the FPLDG group, they are placed in a supine position with legs abducted. A 10 mm Trocar is inserted through a small incision below the umbilicus as the observation port. After exploring the abdominal and pelvic organs to confirm the absence of distant metastasis, a 12 mm Trocar is inserted below the costal margin at the left anterior axillary line as the main operation port, and a 5 mm Trocar is inserted at the umbilical level on the left midclavicular line as the auxiliary port. The placement on the right side is the opposite of that on the left side. The chief surgeon stands on the left side of the patient, the assistant stands on the right side of the patient, and the scope holder stands between the patient’s legs. For the TPTLDG group, on the basis of the control group, the two Trocar ports on the right side (assistant’s position) are removed, and the other layout is consistent with that of the FPLDG group (14).

### 2.4 Surgical management

The technical details of the three-port laparoscopic gastrectomy for GC with the left-side standing position have been described in previous reports (14). Briefly, the patient is placed in a supine position with the head elevated and the feet lowered. An observation port and two operation ports are created at the umbilicus and on the left and right sides of the patient respectively. The operation is performed without the assistance of an assistant. The surgical operation techniques, including gastric resection and lymph node dissection, follow the principles of the guidelines for the treatment of GC, which are consistent with those of traditional laparoscopic gastrectomy for GC. D2 lymph node dissection is performed for all tumors. After the gastric body is transected by a laparoscopic stapler, the 10 mm Trocar port below the umbilicus is enlarged to 3–4 cm to remove the specimen. Depending on the location of the tumor, Billroth-I anastomosis or Billroth-II posterior gastric wall anastomosis is selected, and Braun anastomosis is completed at the umbilical incision. The surgeon evaluates whether to place a drainage tube according to the completion of the operation and records the placement situation. No nasogastric tube is placed in all patients. The urinary catheter is removed immediately after the patient wakes up from anesthesia. For other management, multimodal analgesia, early mobilization, and early oral feeding are carried out with reference to the consensus guidelines of enhanced recovery after surgery for GC.

### 2.5 Main observation indicators

Postoperative inflammatory and nutritional indicators: preoperative and postoperative levels of high-sensitivity C-reactive protein (hs-CRP), interleukin-6 (IL-6), white blood cell count (WBC), and albumin (Alb).

Postoperative complication rate and mortality: defined as any complication or death occurring within 30 days postoperatively. The severity of complications was classified according to the Clavien-Dindo grading system (15).

Long-term survival outcomes: follow-up was conducted via hospitalization records, outpatient visits, WeChat, or telephone to assess complications within 30 days postoperatively, as well as 30-day and 90-day readmission rates. Follow-up continued until March 2025.Overall survival (OS): Defined as the time from surgery to death from any cause. Disease-free survival (DFS): Defined as the time from surgery to death or disease recurrence. Postoperatively, patients were followed up every 6 or 12 months for 5 years, including chest and abdominal computed tomography (CT) and endoscopic examinations. Patients with pathological stage II-III received guideline-recommended chemotherapy regimens.

### 2.6 Statistical methods

Normally distributed continuous variables were expressed as mean ± standard deviation ( $\bar{\text{x}}$ ± s) and compared using Student’s t-test. Categorical variables were compared using the χ² test or Fisher’s exact test, as appropriate. Non-normally distributed continuous variables were expressed as median (Q1, Q3) and compared using the Mann-Whitney U test. Count data were presented as absolute numbers. Kaplan-Meier analysis was used to assess survival rates. All statistical analyses were performed using SPSS 22.0, and a P-value < 0.05 was considered statistically significant.

## 3 Results

### 3.1 Pathological characteristics and operative outcomes

No significant differences were observed between the two groups regarding age, BMI, sex, ASA classification, tumor stage, histological type, or Lauren classification (**Table 1**). The operative time was 140 (125, 160) minutes in the TPLDG group versus 135 (120, 150) minutes in the FPLDG group (*p*=0.068). Estimated blood loss was 80 (50, 100) mL and 100 (125, 160) mL, respectively(*p*=0.128). The numbers of total retrieved lymph nodes were 22(19,27) in the TPLDG group and 22(18,27)in the FPLDG group (*p*=0.696).The two groups showed comparable results in postoperative complication rates, mortality, and 30-/90-day readmission rates. (**Table 2**).

**Table 1:** Demographic and clinical characteristics of the study population.

Baseline date	TPLDG group (n = 183)	FPLDG group (n = 172)	Statistical value	*p*-value
Sex			*F*=0.141	0.708
Male	106 (57.9%)	103 (59.9%)		
Female	77 (42.1%)	69 (40.1%)		
Age, median, (IQR), (year)	55.84 ± 10.26	55.65 ± 10.44	*Z*=0.173	0.863
BMI, Median (IQR), (kg/m2)				
Median [Q1, Q3]	20.7 (19.6,21.9)	20.8 (19.3,21.5)	*Z*=-0.152	0.879
Mean (SD)	20.71 ± 1.46	20.70 ± 1.36	*t*=0.045	0.964
ASA scores			*F*=0.603	0.740
I	121 (66.1%)	111 (64.5%)		
II	51 (27.9%)	53 (30.8%)		
III	11 (6.0%)	8 (4.7%)		
Histologic type			*F*=3.284	0.194
Well differentiated	54 (29.5%)	50 (29.1%)		
Moderately differentiated	54 (27.9%)	35 (20.3%)		
Poorly differentiated	78 (51.5%)	87 (50.6%)		
Lauren classification			*F*=2.408	0.492
Intestinal	63 (34.4%)	70 (40.7%)		
Diffuse	68 (37.2%)	64 (37.2%)		
Mixed	41 (22.4%)	29 (16.9%)		
Unclassified	11 (6.0%)	9 (5.2%)		
Depth of invasion			*F*=0.817	0.057
pTis	3 (1.6%)	5 (2.9%)		
pT1	3 3 (18.0%)	24 (14.0%)		
pT2	59 (32.2%)	65 (37.8%)		
pT3	70 (38.3%)	62 (36.0%)		
pT4a	18 (9.8%)	16 (9.3%)		
Nodal metastasis			*F*=0.936	0.817
pN0	99 (54.1%)	90 (52.3%)		
pN1	45 (24.6%)	44 (25.6%)		
pN2	25 (13.7%)	28 (16.3%)		
pN3	14 (7.7%)	10 (5.8%)		
pTNM stage			*F*=1.349	0.717
0	3 (1.6%)	5 (2.9%)		
I	55 (30.1%)	51 (29.7%)		
II	90 (49.2%)	89 (51.7%)		
III	35 (19.1%)	27 (15.7%)		

**Table 2:** Surgical and postoperative recovery characteristics of the study population.

Item	TPLDG group (n = 183)	FPLDG group (n = 172)	F/t/Z value	*p*-value
Operative time (min)				
Median [Q1, Q3]	140 (125,160)	135 (120,150)	*Z*=-1.823	0.068
Estimated blood loss (mL)				
Median [Q1, Q3]	80 (50,100)	100 (125,160)	*Z*=-1.521	0.128
Total LN retrieved [IQR]				
Median [Q1, Q3]	22 (19,27)	22 (18,27)	*Z*=-0.390	0.696
Metastatic LN [IQR]				
Median [Q1, Q3]	1 (0,5)	2 (0,5)	*Z*=-0.628	0.530
Tumor size (cm)				
Median [Q1, Q3]	2.8 (1.9,3.6)	2.5 (2.0,3.7)	*Z*=-0.567	0.571
Proximal margin (cm)				
Median [Q1, Q3]	4.7 (4.1,5.3)	4.6 (4.1,5.3)	*Z*=-0.667	0.504
Time to first ( $\bar{\text{x}}$ ± s) (d)				
Aerofluxus				
Median [Q1, Q3]	2 (2,3)	3 (2,3)	*Z*=-4.067	<0.001
Defecation				
Median [Q1, Q3]	3 (3,4)	3 (3,4)	*Z*=-0.156	0.876
Liquid diet				
Median [Q1, Q3]	2 (2,2)	2 (2,3)	*Z*=-0.013	0.990
Hospital stay (day)				
Median [Q1, Q3]	4 (3,5)	5.2 (4.2,6.3)	*Z*=-7.719	<0.001
Anastomosis method			*F*=1.307	0.253
Billroth I	23 (12.6%)	29 (16.9%)		
Billroth II	160 (87.4%)	143 (83.1%)		
Tubeless			*F*=35.098	<0.001
Yes	79 (43.2%)	25 (14.5%)		
No	104 (56.8%)	147 (85.5%)		
Retention Duration of the Drainage Tube (day, Mean ± SD)	2.05 ± 0.96	3.03 ± 1.16	*t*=-7.176	<0.001
Morbidity				
Grade I-II	7 (3.8%)	9 (5.2%)	*F*=0.408	0.523
Grade III	2 (1.1%)	3 (1.7%)	*F*=0.272	0.603
30-day unplanned readmission			*F*=0.487	0.485
Yes	5 (2.7%)	7 (4.1%)		
No	178 (97.3%)	165 (95.9%)		
90-day unplanned readmission			*F*=0.769	0.381
Yes	15 (2.7%)	10 (4.1%)		
No	168 (91.8%)	162 (94.2%)		
Total hospitalization cost				
Median [Q1, Q3]	68678.80 (58220.41,78576.94)	74913.11 (63791.11,85691.71)	*Z*=-3.921	<0.001

### 3.2 Recovery outcomes

The TPLDG group showed earlier time to first flatus[2(2,3) *vs.* 3(2,3) day, *Z*=-4.067, *p*<0.001], shorter hospital stay [4(3,5) *vs.* 5.2(4.2,6.3) day, *Z*=-7.719, *p*<0.001]), higher tubeless rate, and lower total hospitalization costs in terms of postoperative recovery.(**Table 2**)To further compare the differences in postoperative recovery between the two groups, we analyzed inflammatory markers on postoperative day 3. The results demonstrated that the TPLDG group had significantly lower levels of C-reactive protein (CRP) and interleukin-6 (IL-6) compared to the FPLDG group (**Table 3**).

**Table 3:** Comparison of inflammatory response markers and nutritional indicators between the two groups.

Baseline date	TPLDG group (n = 183)	FPLDG group (n = 172)	*t*-value	*p*-value
Preoperative				
Albumin ( $\bar{\text{x}}$ ± s) (g/L)	43.57 ± 3.06	41.57 ± 5.49	1.705	0.096
C-Reactive Protein( $\bar{\text{x}}$ ± s) (mg/L)	3.05 ± 1.97	4.84 ± 2.29	-0.889	0.378
IL⁃6( $\bar{\text{x}}$ ± s) (pg/mL)	8.43 ± 2.39	9.67 ± 3.79	-1.373	0.175
White Blood Cell count( $\bar{\text{x}}$ ± s) (×10^9^/L)	6.44 ± 2.22	6.46 ± 2.90	-0.022	0.982
Postoperative				
Albumin ( $\bar{\text{x}}$ ± s) (g/L)	36.08 ± 4.62	34.14 ± 7.68	1.190	0.239
C-Reactive Protein( $\bar{\text{x}}$ ± s) (mg/L)	5.52 ± 2.20	11.14 ± 4.81	-1.952	0.041
IL⁃6( $\bar{\text{x}}$ ± s) (pg/mL)	28.57 ± 18.99	49.69 ± 30.54	-2.518	0.016
White Blood Cell count( $\bar{\text{x}}$ ± s) (×10^9^/L)	9.64 ± 3.12	10.08 ± 5.55	-0.377	0.708
Pre-Post difference				
Albumin ( $\bar{\text{x}}$ ± s) (g/L)	-7.49 ± 4.23	-7.45 ± 8.41	-0.034	0.973
C-Reactive Protein( $\bar{\text{x}}$ ± s) (mg/L)	2.48 ± 6.54	10.29 ± 5.99	-1.097	0.021
IL⁃6( $\bar{\text{x}}$ ± s) (pg/mL)	20.14 ± 17.7	34.57 ± 16.70	-1.538	0.033
White Blood Cell count( $\bar{\text{x}}$ ± s) (×10^9^/L)	3.20 ± 3.46	3.62 ± 6.52	-0.314	0.755

### 3.3 Survival outcomes

**Figure 2:**
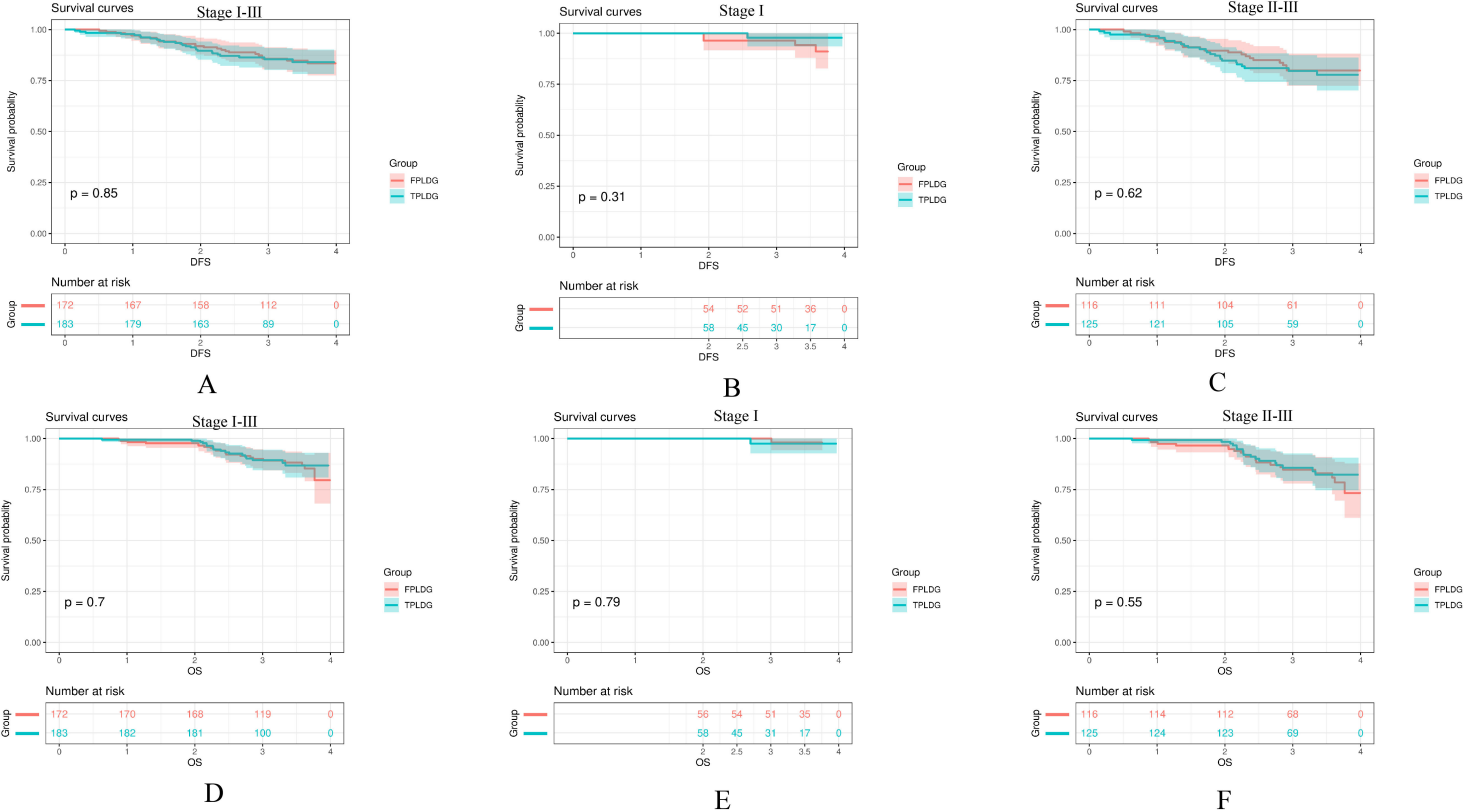
Kaplan-Meier survival curves comparing the TPLDG and FPLDG groups: **(A)** Disease-free survival (DFS), **(B)** DFS in stage I, **(C)** DFS in stages II–III, **(D)** Overall survival (OS), **(E)** OS in stage I, and **(F)** OS in stages II–III.

The 3-year DFS rates were 85.4% for the TPLDG group and 85.8% for the FPLDG group (*p*=0.85, **Figure 2A**), while the 3-year OS rates were 89.4% and 89.2%, respectively (*p*=0.70, **Figure 2D**). Given the significant influence of disease stage on prognosis, we performed a stratified survival analysis. As shown in **Figures 2B, C, E, F**, the survival curves were compared between the two groups across different pathological stages. No significant differences in DFS were observed in either stage I (*p*=0.31, **Figure 2B**) or stages II–III (*p*=0.62, **Figure 2C**). Similarly, OS did not differ significantly between the groups for stage I (*p*=0.79, **Figure 2E**) or stages II–III (*p* = 0.55, **Figure 2F**).

## 4 Discussion

With the continuous development of surgical techniques and instruments, reduced-port laparoscopic GC surgery has gradually received attention in clinical practice (8, 10, 13). This study focuses on left-side standing three-port laparoscopic radical distal gastrectomy, exploring its long-term safety and efficacy, and has certain significance in the treatment of early and some advanced GC.

Regarding the application status of reduced-port techniques, the single-port method has gradually been replaced by the single-port +1 technique (16–18). However, it requires an additional port, increasing costs, and also poses problems such as interference between instruments and limited visual fields (19, 20). The long-term safety of the right-side standing three-port method has been verified, but it is limited to early GC, and there are certain difficulties in D2 lymph node dissection (8). In comparison, the TPTLDG in this study shows unique advantages. It does not require special equipment. The design of the two ports effectively avoids the problems of instrument conflicts and visual field limitations in single-port +1 surgery. The standing position of the surgeon and the surgical operation are similar to those of traditional laparoscopic surgery, making it easier for gastrointestinal surgeons to master (14). In terms of effectiveness and safety, this study has been verified by prospective data. In patients with early and advanced GC, the long-term survival rate of TPLDG is equivalent to that of FPLDG, and it does not increase the risk of surgical complications. This result is consistent with other studies. The main reason is that the cases are mainly early and mid-stage GC, and there is no significant difference in the number of lymph nodes dissected.

The results of our previous studies have shown that patients in the TPTLDG group have shorter incision lengths and smaller umbilical incisions, reducing abdominal wall trauma and improving the aesthetic effect of the abdominal wall (14). In terms of postoperative recovery-related indicators, the overall levels of CRP and IL-6 on the third day after surgery in the TPTLDG group are lower, indicating that patients have a smaller stress response to reduced-port surgery (20). This also leads to a shorter time for the first postoperative anal exhaust and a shorter hospital stay (7). All these indicate that TPTLDG is helpful for patients’ postoperative recovery. We believes that this is because reduced-port surgery requires more precise and accurate operation, and it is mostly carried out by experienced surgeons, reducing repeated grasping of tissues (7, 21). Compared with FPLDG, the TPLDG group has no repeated grasping of tissues by assistants, which may reduce tissue damage to a certain extent. From the perspective of health economics, TPTLDG reduces the instruments used by assistants, does not require additional puncture devices and professional multi-channel puncture devices, reduces the cost of instruments, and has a shorter hospital stay, so the hospitalization cost is lower (22).

In addition, this study also summarizes the operation key points of TPTLDG: an independent main surgeon operation port, the left-side standing position is more in line with the traditional standing position of the surgeon, and in special cases, Trocar can be added to convert to FPLDG; through the left-side approach, it is safe and feasible to perform D2 lymph node dissection on some patients with advanced gastric cancer and those who have received neoadjuvant chemotherapy; during gastroenteric anastomosis, the surgeon needs to skillfully cooperate with both hands and use a laparoscopic linear cutting and closing device to complete the operation; after the surgeon learns the reduced-port surgical method, the concept of simplifying complexity established can help guide conventional laparoscopic surgery.

However, this study also has certain limitations. In terms of the research design, although it is a prospective study, it uses an observational design, making it difficult to completely avoid selection bias. The surgeons participating in the study are all experienced gastric surgery experts, but the factor of the learning curve has not been fully considered, which makes it necessary to be cautious when promoting the research conclusions. In addition, the clinical trial only included patients with distal gastric cancer and those with a BMI ≤ 24 kg/m², and did not involve total gastrectomy and proximal gastrectomy, because the lymph node dissection and digestive tract reconstruction of the latter two are more difficult, and it is more prudent to carry out relevant trials while ensuring surgical safety.

In conclusion, this study confirms that for patients with early and some advanced GC, TPTLDG surgery has equivalent long-term oncological efficacy to traditional five-port surgery, and has the advantages of short abdominal wall incisions, small surgical trauma, fast postoperative recovery, and low hospitalization costs. Under the condition of ensuring patient safety, it can be used as an effective solution to the shortage of surgical assistants. However, due to the limitations of the study, large-scale randomized clinical trials are still needed in the future to further clarify its clinical advantages in patients with different stages of gastric cancer and different BMIs, and to promote the development and application of reduced-port laparoscopic gastric cancer surgery.

## Data Availability

The datasets presented in this article are not readily available because of the need to protect patient privacy data. Requests to access the datasets should be directed to JW, weijiangpeng2015@163.com.
